# Sludge amendment accelerating reclamation process of reconstructed mining substrates

**DOI:** 10.1038/s41598-021-81703-9

**Published:** 2021-02-03

**Authors:** Dan Li, Ningning Yin, Ruiwei Xu, Liping Wang, Zhen Zhang, Kang Li

**Affiliations:** 1 School of Environment and Spatial Informatics, China University of Mining and Technology, Xuzhou, 221116 China; 2 School of Chemistry and Environmental Engineering, Jiujiang University, Jiujiang, 332005 China; 3 College of Environmental Sciences and Engineering, Peking University, Beijing, 100871 China; 4 Engineering Research Center of Mine Ecological Construction, Ministry of Education, Xuzhou, 221116 China; 5 College of Natural Resources and Environment, Joint Institute for Environmental Research and Education, South China Agricultural University, Guangzhou, 510642 China

**Keywords:** Ecology, Environmental sciences

## Abstract

We constructed a mining soil restoration system combining plant, complex substrate and microbe. Sludge was added to reconstructed mine substrates (RMS) to accelerate the reclamation process. The effect of sludge on plant growth, microbial activity, soil aggregate stability, and aggregation-associated soil characteristics was monitored during 10 years of reclamation. Results show that the height and total biomass of ryegrass increases with reclamation time. Sludge amendment increases the aggregate binding agent content and soil aggregate stability. Soil organic carbon (SOC) and light-fraction SOC (LFOC) in the RMS increase by 151% and 247% compared with those of the control, respectively. A similar trend was observed for the glomalin-related soil protein (GRSP). Stable soil aggregate indexes increase until the seventh year. In short, the variables of RMS determined after 3–7 years insignificantly differ from those of the untreated sample in the tenth-year. Furthermore, significant positive correlations between the GRSP and SOC and GRSP and soil structure-related variables were observed in RMS. Biological stimulation of the SOC and GRSP accelerates the recovery of the soil structure and ecosystem function. Consequently, the plant–complex substrate–microbe ecological restoration system can be used as an effective tool in early mining soil reclamation.

## Introduction

The ecological restoration of coal mining areas is strongly limited by soil nutrient deficiencies, unstable soil structures, and slow growth rates of the vegetation. Soil reconstruction is the core component of land reclamation, and thus the quality of reconstructed soil directly determines the quality of land reclamation^1^. Coal gangue and fly ash are produced during coal mining, processing, and use. In recent years, an increasing amount of research has begun to explore the use of industrial solid waste in mining areas to build reconstructed mine substrates (RMS), such as coal gangue^2,3^. On this basis, sewage sludge is one of the amendments used to enrich RMS and speed up the reclamation process. Positive effects of sludge amendment have been reported in many studies. For example, sludge amendment enriches the soil nutrients and promotes plant growth^4–6^. Sludge amendment is an important factor stimulating the microbial activity and development of microbial communities in the soil.

Soil microbial communities, such as bacteria and fungi, play vital roles in nutrient cycling, reduction in plant stresses, and formation of soil aggregates^7,8^. Arbuscular mycorrhizal fungi (AMF) are abundant in various types of soils and are particularly critical for the improvement of the plant growth and adaptation to stressed environments^9^. AMF transfer mineral nutrients to the host plants. In return, plants provide lipids and sugars to their fungal partners^10^. Glomalin is a typically heat resilient soil glycoprotein, which is produced by AMF during symbiotic association with the roots of about 70% of all plant families^11,12^. The quantification of glomalin is often tagged as glomalin-related soil protein (GRSP)^13,14^. To follow AMF development over longer time scales, the determination of GRSP as a stable, specific biomarker has been suggested^15^. It has been hypothesised that the glomalin acts as “glue” during the formation of the aggregate and substantially contributes to the soil organic carbon (SOC)^14^.

As an essential indicator of the soil quality, the SOC and its different labile fractions play important roles in determining the chemical, physical, and biological properties of RMS. Most previous studies focused on total carbon sequestration and the results showed that the SOC increases with the reclamation time^16,17^. The SOC consists of various components with different physicochemical properties, degrees of stabilisation, and turnover rates. The light-fraction SOC (LFOC) content is primarily derived from plant residues, roots, and fungal hyphae in different decomposition stages and is generally sensitive to land-use changes, plant types, and soil depths^18^. The distribution of the different functional SOC pools plays a key role in the SOC accumulation^19,20^.

Soil organic matter is a source of energy and carbon for soil microorganisms, such as bacteria and fungi, which in turn enhance the formation of soil micro- and macroaggregates through mucilage^21,22^. Therefore, the aggregate stability is a widely used indicator for the evaluation of the physical soil quality and susceptibility to erosion^23^. Researchers have emphasised the interrelation between the GRSP, SOC, and different sources of compost and sewage sludge^24^. However, there is a lack of data regarding the accumulation of the GRSP and SOC during reclamation. The strong interrelationship among the SOC, GRSP, and soil aggregation might be due to organic amendment. Long-term monitoring of the soil quality during reclamation is vital for gaining insights into the soil improvement level.

We reconstructed a plant–complex substrate–microbe ecological restoration system in the Pangzhuang coal mine in the northeast of Xuzhou, China, and sludge was added to the RMS to accelerate the reclamation process. The objectives during the ten-year reconstruction were as follows: (i) investigate the response of the plant development and soil microbial activity to sludge amendment in RMS; (ii) assess the effect of sludge amendment on SOC and SOC fraction (LFOC) and GRSP (easily extractable GRSP, EE-GRSP; total GRSP, T-GRSP); and (iii) identify the interrelationship among the SOC, GRSP, and soil aggregation.

## Results

### Growth of ryegrass

Table 1 presents the height and biomass of ryegrass in RMS. Significant differences were observed between the tested treatments. The height increases with the reclamation time and eventually is 1.5 times higher than that of CK2 (i.e. control group 2; see “Materials and methods”) in the tenth-year reclamation. The changes of the aboveground biomass and root biomass of ryegrass are similar to that of the height in RMS; they reach a maximum of 19.58 × 10^–2^ g m^−2^ and 2.15 × 10^–2^ g m^−2^, respectively, in the tenth year. The growth of ryegrass in RMS exceeds that of CK10 (i.e. control group 10; see “Materials and methods”) in the sixth year. Height and biomass of ryegrass remain increasing throughout the whole experiment period while the total biomass of the ryegrass progressively increases.

**Table 1:** Height and biomass of ryegrass in reconstructed mine substrates (RMS).

Ryegrass	2 years	4 years	6 years	8 years	10 years	CK2	CK10
Height (cm)	54 (1.05)	55 (0.72)	58 (0.58)	60 (1.35)	61 (1.29)	41 (1.24)	56.12 (1.01)
Aboveground biomass (*10^–2^ g m^−2^)	17.41 (2.52)	18.01 (1.02)	18.55 (1.01)	19.53 (1.04)	19.58 (1.21)	5.79 (0.78)	18.32 (0.58)
Root biomass (*10^–2^ g m^−2^)	1.49 (0.22)	1.61 (1.03)	2.07 (0.12)	2.14 (0.1)	2.15 (0.11)	0.65 (0.09)	2.05 (0.12)

Parentheses show standard deviations.

### Effect of sludge addition on the functional diversity of the microbial community in RMS

**Figure 1:**
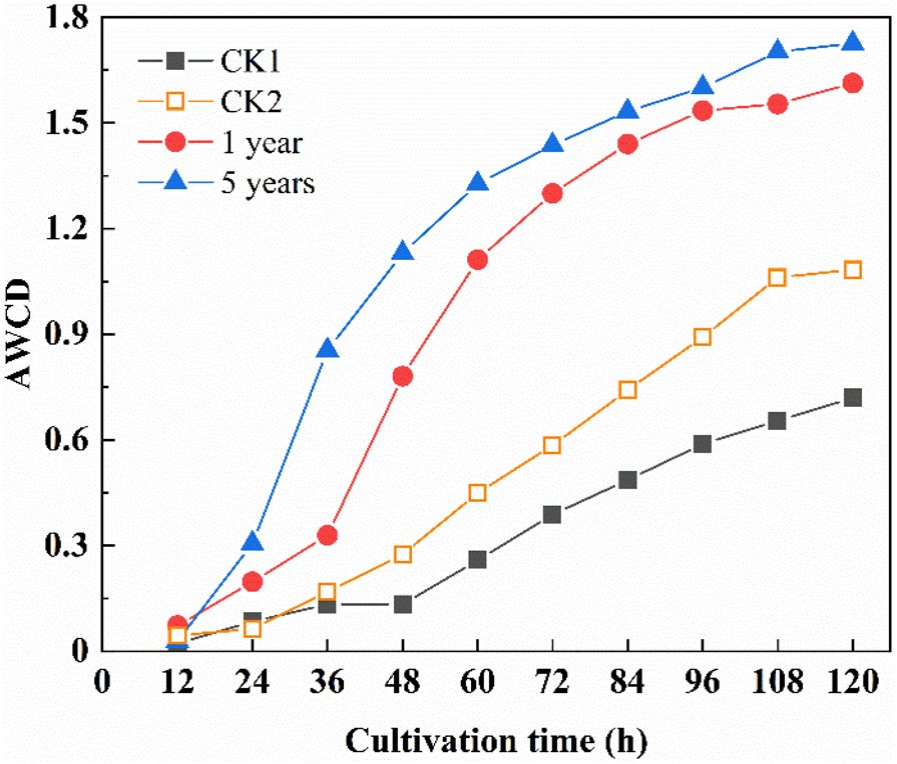
Average well colour development (AWCD) of the microbial community in reconstructed mine substrates (RMS) for different years.

Figure 1 shows that the average well colour development (AWCD) of the microbial community in the reclaimed mine areas is of “S” type, with the culture time both in the first and fifth years. In the initial stage of reclamation, the AWCD values of the four groups are below 0.3 and insignificantly change within 24 h. The AWCD values then rapidly increase, indicating that the microorganisms have an enhanced ability to utilise the C sources during 24–48 h. Finally, the growth almost stops after 120 h. The AWCD values of the four reclaimed substrates change as follows: slow growth, rapid growth, and stabilisation. This indicates that the microorganisms exhibit strong metabolic activities after a period of adaptation. Both in the first and fifth years, the AWCD values are significantly higher than those of CK1 (i.e. control group 1; see “Materials and methods”)and CK5 (i.e. control group 5; see “Materials and methods”) and stabilise at 1.61 and 1.73, respectively. This study shows that the microbial activity in the fifth year is higher than that in the first year. The results suggest that the microbial activity increases by sludge addition with the RMS age.

We also analysed the AWCD data of the microplates cultured for 120 h to compare the functional diversities of the microbial communities in the different RMS. Table 2 shows that each mine substrate of the four groups has the same Simpson index (D). In contrast, the corresponding Shannon (H) and McIntosh (U) indices significantly differ. The H value in RMS is higher than that in CK1 and CK5 in the first year, but there is no difference between the first and the fifth years, implying that the metabolic activity of the microbial community peaks in the first year both in the CK (i.e. control groups; see “Materials and methods”) and RMS groups. The U values in RMS increase with the reclamation time after 120 h and are significantly higher than those of CK1. After one year, they seem to remain stable.

**Table 2:** Functional diversity (i.e. Shannon (H), Simpson (D), and McIntosh (U)) indices of the soil microbial community in reconstructed mine substrates (RMS).

Treatment	H	D	U
CK1	3.06 (0.18) a	0.95 (0.01) a	6.08 (1.09) a
CK5	3.07 (0.18) a	0.95 (0.01) a	8.34 (0.95) b
1 year	3.32 (0.02) b	0.96 (0.01) a	10.08 (0.19) c
5 years	3.23 (0.06) ab	0.96 (0.01) a	11.18 (0.59) c

Parentheses show standard deviations. Different small letters indicate significant differences among different reclaimed soil treatments at a significance level of *p* < 0.05.

### Changes of the SOC and GRSP fractions in RMS

**Figure 2:**
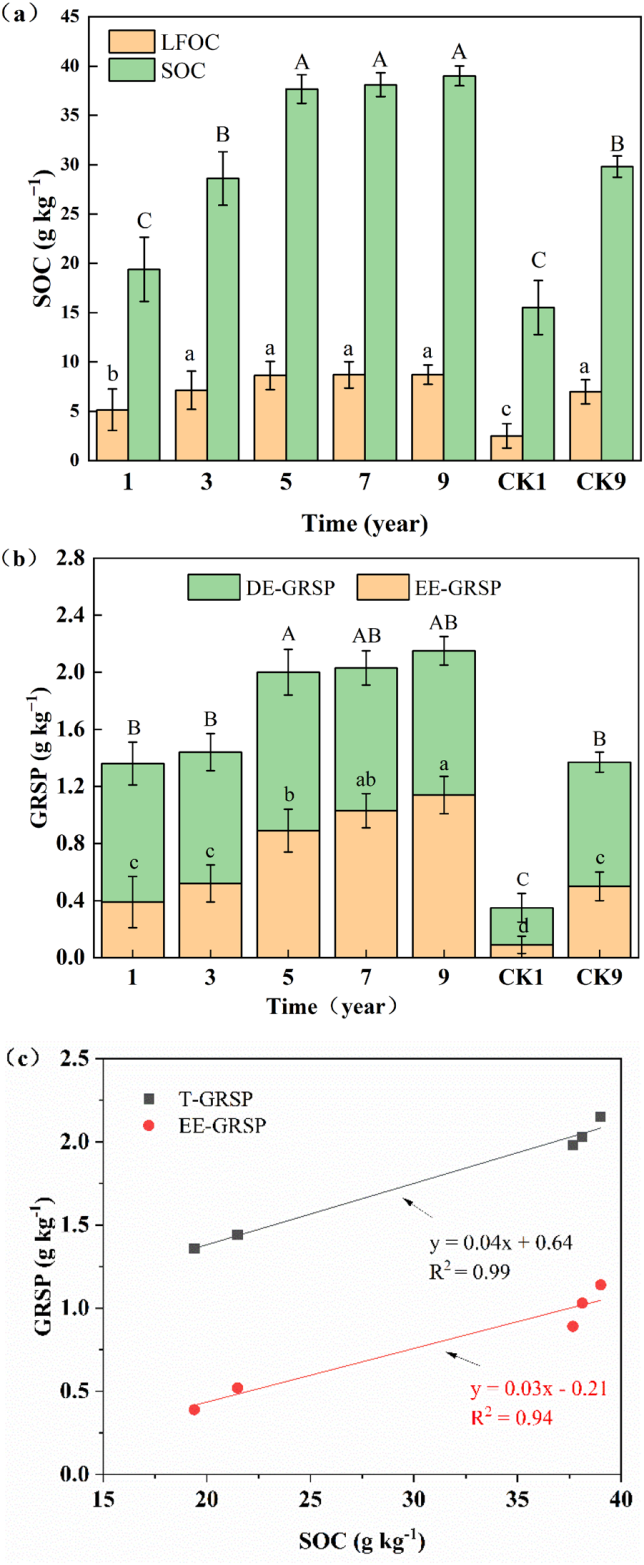
The influence of sludge on (**a**) soil organic carbon (SOC), (**b**) glomalin-related soil protein (GRSP) during the 10-year reclamation period, and (**c**) the relationship between SOC and GRSP under sludge addition. Bars with different letters (lower case for light-fraction SOC (LFOC) and easily extractable GRSP (EE-GRSP), upper case for SOC and difficultly extractable GRSP (DE-GRSP)) indicate a significant difference at the p < 0.05 level. (Total GRSP (T-GRSP) = EE-GRSP + DE-GRSP).

The distribution of different functional pools of SOC and LFOC in RMS significantly increases compared with the CK1 and there is no difference between RMS and CK9 (i.e. control group 9; see “Materials and methods”) since the third year of reclamation (Fig. 2a). The SOC increases in reclaimed areas in which the initial SOC was insufficient. The SOC and LFOC of RMS increase by 151% and 247% with the reclaimed time, respectively, compared with those of CK1. In addition, significant treatment effects (*p* < 0.05) were also observed for the LFOC proportions of the SOC (LFOC/SOC). The LFOC/SOC is the highest after one year (27%), while the lowest value was observed in the CK1 (16%). Hao et al.^25^ showed that the more exogenous organic matter is added, the higher is the SOC content including the LFOC. Sludge addition increases the nutritive element content in the soil and the quantity and activity of soil microorganisms, which accelerates the decomposition of organic matter, especially that of LFOC.

A similar trend was observed for the GRSP pools (Fig. 2b). The EE-GRSP and T-GRSP contents of the studied RMS range from 0.39 to 1.14 g kg^−1^ and from 1.36 to 2.15 g kg^−1^, respectively. The values of these GRSP pools increase with the RMS age and are higher than those of CK1. There is no difference between the GRSP pools of RMS and CK9 in the first and third reclamation. A trend opposite to that of LFOC/SOC was observed for EE-GRSP/T-GRSP, which increases with the RMS age in the third year, with a maximum of 47%. This increase occurs because sludge addition increases the amount of AMF in the soil, which secretes T-GRSP.

We determined a positive linear correlation between glomalin and SOC with sludge addition (Fig. 2c). Both the T-GRSP and EE-GRSP increase with increasing SOC. It has been reported that the SOC and GRSP are closely related in some RMS. Among total SOC components, glomalin accounts for a relatively large proportion of SOC (2–15 mg g^−1^ and contributes to an average 5–10% of total SOC)^26^. Glomalin changes at urban–rural gradients, including urban–rural gradients, urban history gradient and land-use gradient, have been reported that T-GRSP:SOC varied from 10 to 35%^27^. In our experiment, we guess that the larger GRSP:SOC ratio is related to the addition of sludge, the nutrition and structure of the substrate, the infestation of AMF, the reclamation time, and plant growth.

### Improvement of the soil structure in RMS

In general, the agglomerates > 0.25 mm (macroaggregates) form the best structure in the soil. The greater the proportion of agglomerates is, the higher is the stability of the soil. Table 3 shows that the macroaggregate concentration in RMS increases with increasing reclamation time compared with that of CK1, except for the first year. The percentage of aggregates > 0.25 mm reaches 69% after eight-year reclamation, while the mean weight diameter (MWD) and geometric mean diameter (GMD) are 0.63 and 0.47 mm, respectively. Furthermore, the percentage of aggregates > 0.25 mm, MWD, and GMD of RMS in the fifth and seventh years insignificantly differ from those of CK9.

**Table 3:** Effects of organic amendments on the structural stability in reconstructed mine substrates (RMS).

Indicator	1 year	5 years	7 years	9 years	CK1	CK9
> 0.25 mm aggregate (%)	36 (1.21) a	53 (1.42) b	65 (1.25) c	69 (2.02) c	39 (1.32) a	65 (1.23) c
MWD (mm)	0.34 (0.01) a	0.56 (0.01) b	0.61 (0.01) b	0.63 (0.02) b	0.34 (0.01) a	0.61 (0.02) b
GMD (mm)	0.23 (0.01) a	0.36 (0.01) b	0.44 (0.01) c	0.47 (0.01) c	0.23 (0.01) a	0.44 (0.01) c

Parentheses show standard deviations. Dissimilar letters indicate significant differences, whereas similar letters indicate no significant difference among the treatments in the same column at *p* < 0.05.

**Figure 3:**
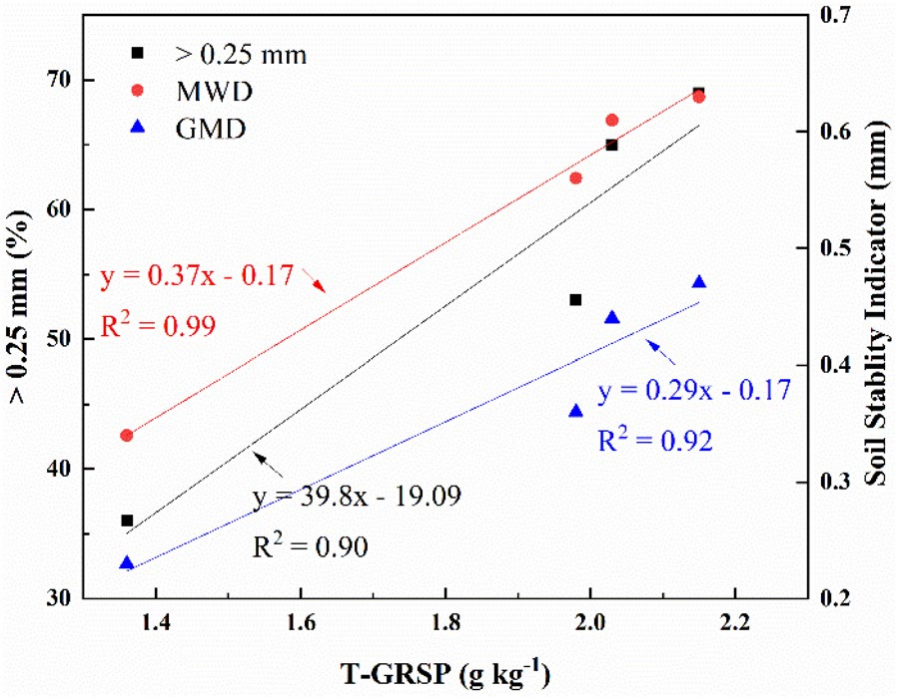
Correlations between the glomalin-related soil protein (GRSP) and aggregate stability indicators during 10-year reclamation.

The GRSP is positively correlated with the aggregate (> 0.25 mm), MWD, and GMD when the sludge is amended (Fig. 3). The macroaggregate content significantly increases and the percentage of aggregates > 0.25 mm, MWD, and GMD increase with the increase in the GRSP content during nine-year reclamation. This study also shows that the percentage of aggregates > 0.25 mm, MWD, and GMD in RMS are significantly higher than those of the untreated substrates.

## Discussion

### Interaction of plant growth and microbial diversity

In the present study, a positive effect of sludge amendment on plant growth was observed in terms of the ryegrass height and biomass at early stage (Table 1). At the same time, the microbial activity increases with the RMS age, while the dominance of the microorganisms of the RMS and CK groups insignificantly changes. The results agree with those of previous chronosequence studies that were conducted in reclaimed mine spoil dumps at other locations^28,29^.

We have explored the changes of microbial diversity at different times after the disturbance in the earlier stage. In fact, the microbial diversity changed greatly within 120 h after adding sludge and reached a relatively stable state after 120 h. We chose 120-h data for follow-up research considering our observation time was 10 years. Different positive effects of sludge on microorganisms in the soil have been reported, including improvement of soil structure and soil stabilisation, soil enrichment with phosphorus and other elements, and stimulation of microbial activity^30^. These effects have a positive effect on most microorganisms and are not selective. Therefore, the non-selective positive effects of sludge kept the community richness and dominance stable. The sludge addition is more advantageous for improving the species uniformity of the microbial communities.

AMF are dominant and ubiquitous soil microbes. Available literature suggests that the majority of the plants present in reclaimed mine dumps belong to the AMF category; AMF are conducive to mycorrhizal activity and plant productivity because they improve the availability of nutrients in the soil^31,32^. Therefore, we speculate that this enhancement might be caused by the increase in AMF due to the sludge amendment.

Regarding the source of AMF, we guess that it is of particular importance to the functionality of the symbiosis are the role of soil animals in grazing the external mycelium. Dispersal and predation both result from animals consuming mycorrhizal fungi. Spores disperse through space, and this dispersal can be promoted by animals. Spores can survive ingestion. For example, the Streptomyces were completed life cycle by dispersal of spores by soil arthropods^33^. Specimens of some Blackburnium, Bolboleaus and Bolborhachium species were found to have ingested diffuse glomeralean mycelium and spores along with varying quantities of soil^34^.

The substrates of the control groups and experimental groups were not sterilised, and the experimental environment was in the open air to be closer to the on-site reclamation. We speculate that AMF spores from animals in the surrounding soil slowly infect the ryegrass roots in the test substrates. Therefore, AMF in the test substrates increased with time while the infection rate increased. At the same time, the GRSP content also increased.

In addition to sludge, there are some factors that may affect the growth of AMF in our research. Firstly, weed invasion is a prevailing problem in modestly managed lawns^35^. Other plants invaded the experimental site during the long-term planting process which could affect ryegrass and AMF in the substrates. Secondly, correlations among soil properties, spore densities, and mycorrhizal colonisations were more pronounced in the higher coverage levels^35^. Although annual ryegrass seeds were broadcasted at 20–30 g m^−2^, the actual coverage level was not reached the seeding density. In other words, the actual coverage level of ryegrass increased with time until the fifth year.

### Interaction of SOC, GRSP and soil stability under sludge

As mentioned earlier, to follow AMF development over longer time scales, the determination of GRSP as a stable, specific biomarker has been suggested^15^. Therefore, we conclude that GRSP originated from AFM both in sludge remediation site and control site in our research. This study shows a marked increase in LFOC with increasing RMS age in first 3 years. Then LFOC presumably reaches a saturation point. The functional pools of the GRSP, such as EE-GRSP and T-GRSP, increase with the RMS age. At the same time, EE-GRSP increases in the fifth year whereas DE-GRSP is stable. We speculate that the slow increase in AMF leads to an increase in the lag of EE-GRSP. It may be that AMF produces a lot of EE-GRSP after the fifth year while the agglomerates increase significantly. EE-GRSP is tightly wrapped by the agglomerates, resulting in the low content of LFOC tested and no positive correlation between LFOC and EE-GRSP.

Some researchers promoted the notion that GRSP can be used as an indicator of soil quality^11^. In this study, there is a positive linear correlation between glomalin and SOC (Fig. 2c) while the GRSP is positively correlated with the aggregate (> 0.25 mm), MWD, and GMD when the sludge is amended (Fig. 3). The results indirectly showed that SOC is positively correlated with aggregate stability. This correlation between the fractions of the GRSP and SOC might be due to the GRSP, which is also a component of the soil organic matter. In contrast, the sludge amendment increases the SOC, resulting in the stimulation of sporulation in several AMF and thus in increased GRSP production^36^. The GRSP and SOC more likely depend on similar ecosystem components, such as the photosynthetic productivity, and contribute to similar ecosystem functions such as the feeding of the decomposer community and formation of soil aggregates. Notably, sludge amendment accelerates the functions of the environmental system by promoting biostimulation.

The results confirm that reclamation improves the soil structure with increasing reclamation time. This improvement was expected because organic materials, transient organic binding agents, and root exudates are conducive to soil aggregation with increasing reclamation time. The results of this study show that sludge amendment in addition to fly ash and coal gangue improves plant productivity, suggesting a gain in about 5 years, over a ten-year trial, in contrast to 16 years reported by Ahirwal and Maiti^37^. The accumulation of plant biomass, exudates, and GRSP favours the aggregation of soil particles. The GRSP and SOC are important constituents of soil aggregates and the factors driving the increase in the soil aggregate content.

Generally, soil aggregation in RMS follows the same hierarchical formation model as agricultural soils and undisturbed natural soils^38^. This study shows that the macroaggregates increase with the reclamation time. A potential explanation is that decomposed roots and microorganisms construct smaller particles and subsequently give rise to large and cohesive aggregates, which form the soil aggregation hierarchy. The plant growth is generally considered to be a factor that is strongly related to the formation of agglomerates and penetrating roots can mechanically break up existing aggregates^39^. The RMS have a poor soil structure and the macroaggregate content is limited and easily disrupted. Sludge is conducive to soil agglomeration because the organic matter in the sludge as a type of transient organic binding agent plays an important role in the formation of macroaggregates by binding relatively stable microaggregates.

## Conclusion

This study demonstrates the positive role of sludge amendment in RMS after ten-year restoration. Sludge amendment in early mining soil reclamation provides organic matter and large amounts of LFOC, GRSP, and EE-GRSP. The growth of ryegrass and microbial activity increase with the RMS age. Plant roots break down and large and cohesive aggregates form in nine years. At the same time, the SOC and GRSP increase. Consequently, the reclamation process speeds up by sludge amendment. The SOC, GRSP, and aggregate stability in a terrestrial ecosystem are correlated. A positive linear correlation among the glomalin concentration, SOC, and aggregate stability indicators is demonstrated during ten-year reclamation. Overall, these results highlight that sludge amendment as soil restoration technique strengthens the soil structure and accelerates the ecosystem functions, especially during early recovery. The organic matter in the sludge stimulates the soil with increasing reclamation time. Further studies should be carried out to identify the role of microorganisms in the nutrient cycle and mineralisation of different functional SOC pools to explain the SOC and GRSP accumulation mechanism.

## Materials and methods

### Experimental design

The Pangzhuang coal mine in the northeast of Xuzhou, China, was selected as the experimental site in a typical coal mining area. The annual average precipitation is 860 mm and annual mean temperature is 14℃. The study site contains two plots and two treatments were assessed separately: plants growing in unamended soil (control groups, fly ash:coal gangue = 14:84, w/w) and plants growing in amended soil (experimental groups, fly ash:coal gangue:sludge = 10:60:30, w/w). Coal gangue and fly ash hardly contained water, and the water content of sludge was 60%. The characteristics of the reconstructed substrates can be found in Qian et al.^15^. There are 28.42, 1.61, and 74.03 g kg^−1^ organic matter, 0.65, 0.13, and 12.53 g kg^−1^ total nitrogen, and 11.51, 16.79, and 216.11 g kg^−1^ available phosphorus in coal gangue, fly ash and sludge, respectively. The control groups without sludge of the first, second, fifth, ninth and tenth years were named CK1, CK2, CK5, CK9, and CK10, respectively.

The area of each plot was 5 m^2^. A 20 cm stone dam was installed between the plots. The depth of the complex substrates was 15 cm. Annual ryegrass (*Lolium multiforum* Lam. cv. Tregold) was selected as the test plant because of its strong root system, which is conducive to the observation of the root dynamics in the later stage. Ryegrass seeds were purchased from Bailv International Grass Industry Co., Ltd., Tianjin; the germination rate was 94%. Annual ryegrass seeds were broadcasted (20–30 g m^−2^) in September of the first, third, fifth, seventh, and ninth years in the RMS and the first and ninth years in unamended soil, and the grass was cut after 12 months. Pesticides and fertilisers were not applied to the soil during the growth period of the ryegrass. The biomass was measured based on the dry weights of the shoots and roots. We got the grass at the second, fourth, sixth, eighth, and tenth years in the RMS and the second and tenth years in the control groups.

### Soil sample collection and processing

Soil samples were collected from the RMS in September after 1, 3, 5, 7, and 9 years following a serpentine sampling pattern. Triplicate soil samples were collected from a depth of 0–15 cm and thoroughly mixed. The SOC of the soil was determined using the potassium dichromate volumetric external heating method. Under heating conditions, excess potassium dichromate sulfuric acid solution was used to oxidise SOC and the excess potassium dichromate was titrated with ferrous sulfate standard solution. The SOC was calculated from the amount of potassium dichromate consumed using the oxidation correction coefficient^40^.

Three 20 g samples of dried soil (< 2 mm) were placed in 100 mL centrifuge tubes and 40 mL of NaI solution with a density of 1.4 g cm^−3^ was added. After shaking for 60 min on the shaker, the samples were centrifuged at 3000 r min^−1^ for 10 min. The unprecipitated material and supernatant were poured into a vacuum suction filter for suction filtration. After the above-mentioned steps were repeated three times, the residue on the filter paper was washed several times with a 0.01 mol L^−1^ CaCl_2_ solution until no precipitation occurred after the addition of AgNO_3_. Finally, the LFOC was dried and weighed.

### Extraction and determination of the GRSP

GRSP was extracted from the soil samples and the T-GRSP and the EE-GRSP was determined by quantification Colorimetric with minor modifications^41,42^. Difficultly extractable GRSP (DE-GRSP) was the difference between T-GRSP and EE-GRSP.

For EE-GRSP, 1 g soil samples were suspended in 8 mL 20 mM citrate (pH 7.0) and autoclaved for 30 min at 121 °C followed by centrifuging at 10,000*g* for 5 min and collecting the supernatant. For T-GRSP was removed from 1 g soil with 8 mL 50 mM citrate (pH 8.0) and autoclaved for 1 h at 121 °C. The supernatants were separated by and centrifuged at 10,000*g* for 5 min, followed by four cycles of extraction and centrifugation until the supernatant was almost transparent. All extracts from each soil sample were then pooled. We described GRSP concentration as the protein content per gram of dried soil based on Bradford assay. Bovine serum albumin was used as the reference standard.

### Separation of water-stable soil aggregates

The distribution of the water-stable aggregate (WSA) fraction was measured using a soil aggregate analyser (DM200, Shanghai Dema Co., Ltd., China). The instrument contained several standard-aperture separation sieves. Aggregates with different particle sizes were separated from the soil by vibrating the sieves up and down in the water. We selected 100 g of air-dried soil. After sieving, four WSA fractions (> 2, 2–0.5, 0.5–0.25, and < 0.25 mm) were dried at 55 °C until a constant weight was achieved. The proportions of each fraction to the total soil mass were calculated.

The GMD was calculated using Eq. (1):

1 $$GMD\left({\text{mm}}\right)=\mathit{exp}\left\{\frac{\sum {w}_{i}ln{x}_{i}}{\sum {w}_{i}}\right\},$$

where *x*_*i*_ denotes the mean diameter of the aggregate class, *w*_*i*_ is the percentage of the weight of the corresponding aggregate class of the total aggregate weight.

The MWD was calculated using Eq. (2):

2 $$MWD={\sum }_{1}^{n+{1}}\frac{{r}_{i-{1}}+{r}_{i}}{2}\times {m}_{i},$$

where *r*_*i*_ is the aperture of the *i*th mesh (mm), *m*_*i*_ is the mass fraction of the aggregates remaining on *i*th sieve, and *n* is the number of sieves.

### Determination of soil microbial indicators

The capability of soil microbial communities to utilise a variety of individual carbon sources was assessed using BIOLOG-ECO plates (Biolog, Inc., USA). A total of 10 g of fresh soil sample was added to 100 mL phosphate buffer and cultured in a shaking incubator at 25 °C for 30 min. The sample was then diluted 1000 times to ensure that the optical density (OD) value of the sample after dilution at a wavelength of 420 nm was ~ 0.19. A 150 μL sample of the suspension was inoculated into each well. The plates were incubated at 25 °C in an incubator. The colour development in each well was recorded as OD at 590 nm with an automatic microplate reader (Varioskan Flash, Thermo Scientific, USA) at 12 h intervals for 120 h.

The absorbance values were corrected by subtracting the absorbance of the control well (water only) before the data analysis. The microbial activity of each microplate expressed as AWCD was determined using Eq. (3):

3 $$AWCD=\sum {OD}_{i}/{31}\text{,}$$

where *OD*_*i*_ is the optical density of each well.

The Shannon (H), Simpson (D), and McIntosh (U) indices reflect the richness, dominance, and evenness of microbial species, respectively^43^. Based on data obtained after 120 h, they can be calculated following the methodology reported in Magurran^44^.

### Analytical techniques

Analysis of variance (ANOVA) was performed using Statistical Product and Service Solution (SPSS) software (version 24.0). Significance was accepted at *p* < 0.05 in all cases. Linear regressions and coefficients of determination (r) were used to describe the relationship between the aggregate and SOC as well as the GRSP.
